# Simple high-cell density fed-batch technique for high-level recombinant protein production with *Pichia pastoris*: Application to intracellular production of Hepatitis B surface antigen

**DOI:** 10.1186/1475-2859-8-13

**Published:** 2009-02-10

**Authors:** Chandrasekhar Gurramkonda, Ahmad Adnan, Thomas Gäbel, Heinrich Lünsdorf, Anton Ross, Satish Kumar Nemani, Sathyamangalam Swaminathan, Navin Khanna, Ursula Rinas

**Affiliations:** 1 International Centre for Genetic Engineering & Biotechnology, New Delhi, India; 2 Helmholtz Centre for Infection Research, Braunschweig, Germany; 3 Department of Chemistry, Government College University Lahore, Lahore, Pakistan; 4 Fraunhofer ITEM, Hannover/Braunschweig, Germany

## Abstract

**Background:**

Hepatitis B is a serious global public health concern. Though a safe and efficacious recombinant vaccine is available, its use in several resource-poor countries is limited by cost. We have investigated the production of Hepatitis B virus surface antigen (HBsAg) using the yeast *Pichia pastoris *GS115 by inserting the *HBsAg *gene into the alcohol oxidase 1 locus.

**Results:**

Large-scale production was optimized by developing a simple fed-batch process leading to enhanced product titers. Cells were first grown rapidly to high-cell density in a batch process using a simple defined medium with low salt and high glycerol concentrations. Induction of recombinant product synthesis was carried out using rather drastic conditions, namely through the addition of methanol to a final concentration of 6 g L^-1^. This methanol concentration was kept constant for the remainder of the cultivation through continuous methanol feeding based on the *on-line *signal of a flame ionization detector employed as methanol analyzer in the off-gas stream. Using this robust feeding protocol, maximum concentrations of ~7 grams HBsAg per liter culture broth were obtained. The amount of soluble HBsAg, competent for assembly into characteristic virus-like particles (VLPs), an attribute critical to its immunogenicity and efficacy as a hepatitis B vaccine, reached 2.3 grams per liter of culture broth.

**Conclusion:**

In comparison to the highest yields reported so far, our simple cultivation process resulted in an ~7 fold enhancement in total HBsAg production with more than 30% of soluble protein competent for assembly into VLPs. This work opens up the possibility of significantly reducing the cost of vaccine production with implications for expanding hepatitis B vaccination in resource-poor countries.

## Background

Hepatitis B virus (HBV) infection currently affects about 2 billion people worldwide. HBV, which is transmitted by contact with blood or body fluids of an infected person like the human immunodeficiency virus, is 50–100 times more infectious than the latter. Perinatal HBV infections often tend to become chronic. It is estimated that there are more than 350 million chronic HBV carriers around the globe. Chronic HBV infection which most often leads to liver cirrhosis and cancer kills about a million people each year. HBV infection represents a major human disease and is a serious global public health concern [1,2]. This situation still prevails despite the availability of an efficacious hepatitis B vaccine since the early eighties. The first generation vaccine was based on purified ~22 nm HBsAg-containing particles from the plasma of asymptomatic HBV carriers. Major factors that have precluded this vaccine from mass immunization have been safety concerns, restricted supply and high cost. The advent of a safe recombinant hepatitis B vaccine which has helped to reduce the cost has resulted in the inclusion of hepatitis B vaccination in the national infant immunization schedules of around 160 countries [3]. However, in many of these countries vaccination coverage is less than 80% and there are several countries in which the vaccine has yet to be introduced.

Recombinant hepatitis B vaccine, based on HBsAg is being produced in many developing countries using yeast-based heterologous expression systems. The methylotrophic yeast *Pichia pastoris *offers several advantages from the perspective of vaccine production. It possesses a very strong and tightly regulated methanol-inducible alcohol oxidase 1 (*AOX1*) promoter which can be used to drive high-level expression of recombinant proteins. Like *E. coli *it can be cultured in simple inexpensive media. As the *AOX1 *promoter can be controlled by manipulating the carbon source in the medium, *P. pastoris *cultures can be easily scaled up to large bioreactors. The preference of this yeast for aerobic growth permits it to be cultured in the bioreactor to cell densities exceeding 100 g L^-1 ^cell dry mass (CDM). All these features contribute to high productivity. Being a eukaryote, *P. pastoris *is especially suitable for the production of proteins whose functions rely on correct disulfide bonding. Several disulfide-linked, functional recombinant proteins have been successfully produced using *P. pastoris*. Lastly, the yeast is a non-pathogenic organism and recombinant proteins produced by it will be free of pyrogens (unlike *E. coli *derived proteins), toxins and viral inclusions (unlike mammalian cell culture derived proteins) making them safe for human use. Consequently, this yeast has emerged as a preferred host for the production of functional recombinant proteins [4-7].

The use of *P. pastoris *to produce HBsAg is well-documented [8-13]. Several studies have shown that HBsAg produced by *P. pastoris *assembles into characteristic ~22 nm virus-like particles (VLPs) which are highly immunogenic and capable of eliciting potent neutralizing antibodies. An examination of the available literature reveals a wide variation in the reported volumetric product concentrations of HBsAg by different groups, ranging from 0.012 to 1.0 g L^-1 ^(Table 1). Mostly, investigators have used Mut^S ^(methanol utilization slow) strains of *P. pastoris *created by replacing the host *AOX1 *gene with the *HBsAg *gene under the control of the native *AOX1 *promoter. In general, fed-batch protocols developed for large-scale production of recombinant proteins, including HBsAg, by *P. pastoris*, consist of a batch phase with growth on glycerol as carbon source, followed by a glycerol-limited fed-batch procedure to increase the biomass concentration and derepress the *AOX1 *promoter, and, finally, concluded by the induction phase using different methanol feeding protocols.

**Table 1:** HBsAg production with *P. pastoris*

Strain (copy#)	MeOH (%)	Induction time (h)	VLP (method)^a^	Quantification	Yield (g L^-1^)	Reference
Mut^s ^(1)	0.5	200	+ (RIA; EM)	Western	0.4	[11]
Mut^s ^(8)	0.1	192	+ (E)	Western	1.0	[10]
nd (nd)^b^	nd	nd	Nd	E	0.097	[12]
Mut^s ^(nd)	0.5	96	+ (EM)	nd	nd	[9]
Mut^s ^(nd)	1.0	72	Nd	RPHA^c^	0.0125	[8]
Mut^s ^(nd)	0.1	70	Nd	E	0.32	[13]
Mut^+ ^(nd)	0.25	50	Nd	E	0.35	[13]
Mut^s ^(8)	0.6	90–160	+ (EM)	RP-HPLC	6–7	present study

^a ^The '+' symbol denotes detection of virus-like particles (VLP) using radioimmunoassay (RIA), electron microscopy (EM) or ELISA (E)

^b ^nd indicates no data available

^c ^RPHA: reversed passive hemeagglutination assay

In this work, we present a simple fed-batch protocol developed for high-level production of recombinant proteins by *P. pastoris *and the application of this procedure for the intracellular production of HBsAg. We demonstrate that our simple cultivation process resulted in ~7 fold enhancement in total HBsAg production compared to the highest yields reported so far, with more than 30% being soluble protein, competent for assembly into VLPs.

## Results

**Figure 1:**
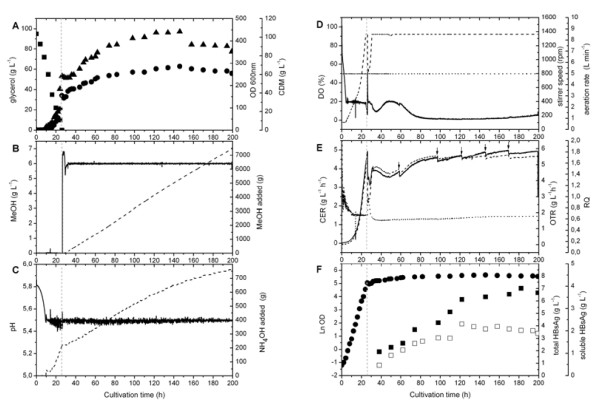
**Two-phase fed-batch cultivation of *P. pastoris *GS115 carrying 8-copies of the *HBsAg *gene under the control of the *AOX1 *promoter**. Cells were first grown in a batch phase with glycerol as carbon source followed by a methanol feeding phase to induce the production of HBsAg. (A) Concentrations of glycerol (filled squares) and biomass (optical density: filled circles; cell dry mass: filled triangles). (B) Concentration of methanol (solid line) and amount of methanol added to the bioreactor (dashed line). (C) Medium pH (solid line) and amount of ammonium hydroxide added to the bioreactor (dashed line). (D) Dissolved oxygen concentration (solid line), aeration rate (dotted line), and stirrer speed (dashed line). (E) Oxygen transfer (dashed line) and carbon dioxide evolution (solid line) rates and respiratory quotient (dotted line). Small arrows indicate removal of culture broth. (F) Cell growth (filled circles) and accumulation of HBsAg (total HBsAg: filled squares and soluble HBsAg: open squares). The dashed vertical line indicates the end of the glycerol batch and the start of the methanol feeding phase.

A simple two-stage fed-batch cultivation technique was adopted for the production of HBsAg using *P. pastoris *GS115 as expression host. The cells were first grown at 30°C (pH 5.5) in a batch procedure using a simple defined medium containing low salt and high glycerol concentration (95 g L^-1^). Using this medium final biomass concentrations of ~60 g L^-1 ^CDM were obtained at the end of the batch phase (Fig. 1A) corresponding to a biomass yield coefficient (Y_X/S_) = 0.61 g g^-1 ^(CDM on glycerol). During the batch phase, a maximum specific growth rate (μ_max_) = 0.17 h^-1 ^was reached. After depletion of glycerol (Fig. 1A), induction of HBsAg production was initiated by the addition of methanol (Fig. 1B). The end of the batch phase was indicated by a sudden increase in the dissolved oxygen (DO) concentration (Fig. 1D) and decrease of the respiratory activity (Fig. 1E). During the fed-batch HBsAg production phase, methanol was the sole carbon and energy source and its concentration was kept constant at 6 g L^-1 ^by continuous feeding based on the signal from a flame ionization detector (FID) employed for *on-line *monitoring of the methanol concentration (Fig. 1B). After the sudden exposure of *P. pastoris *to methanol, biomass concentrations slightly declined but cell growth was resumed after a short period of adaptation (~3–4 h, Fig. 1A). Successful adaptation to methanol was also noticeable by the onset of automated methanol feeding (Fig. 1B), the resumption of ammonia uptake (Fig. 1C), the increase in the respiratory activity (Fig. 1E), and the increased stirrer speed in response to the elevated oxygen demand (Fig. 1D). After the onset of methanol feeding, biomass concentrations increased from 60 g L^-1 ^to ~100 g L^-1 ^CDM after 90–100 h of growth on methanol. Further extension of the fed-batch phase did not result in more growth but led to growth cessation and finally to cell lysis as indicated by decreasing biomass concentrations (Fig. 1A). Moreover, cell viability remained high during the first 90–100 h of growth on methanol (> 90%), but declined considerably beyond (data not shown).

**Figure 2:**
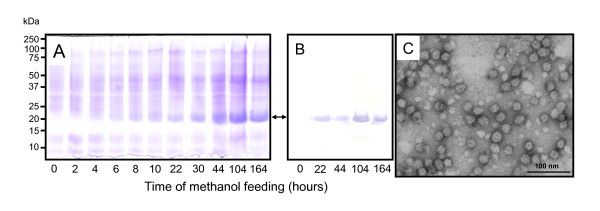
**Time-course analysis of HBsAg production**. (A) Total cell lysates analyzed by SDS-PAGE from samples taken directly before the addition of methanol (time 0) and 2, 4, 6, 8, 10, 22, 30, 44, 104, and 164 hours after the onset of methanol feeding. (B) Western blot analysis of total cell lysates taken directly before the addition of methanol (time 0) and 22, 44, 104, and 164 hours after the onset of methanol feeding using a commercially available rabbit anti-HBsAg antiserum. The bidirectional arrow between panels A and B denotes the position of the HBsAg. (C) Transmission electron microscopic survey of negatively stained, purified HBsAg particles.

Recombinant HBsAg production was analyzed during the induction phase from culture aliquots withdrawn at various time points. The analysis of total cell lysates by SDS PAGE and Western blot demonstrated that HBsAg accumulated as the most prominent protein band of the total cell protein (Fig. 2). Quantification of HBsAg production by RP-HPLC revealed that total HBsAg concentrations reached a maximum of 7 g L^-1 ^(Fig. 1F). The productivity was highest during the first 90–100 h of growth on methanol. Continuation of the induction phase resulted only in a slight increase in the volumetric concentration of total HBsAg. The amount of soluble HBsAg competent for VLP assembly represented about 30% of total HBsAg reaching highest concentrations of 2.3 g L^-1 ^after 90–100 h of growth on methanol (Fig. 1F). Again, extending the methanol feeding phase beyond 90–100 h did not increase the amount of soluble HBsAg but caused a slight decline in the concentration of HBsAg competent for VLP assembly. Finally, analysis of negatively stained, purified HBsAg by electron microscopy revealed the appearance of characteristic VLPs (Fig. 2), a prerequisite of HBsAg applicability for human vaccination against Hepatitis B [11,14-16].

## Discussion

Currently, most *P. pastoris *fed-batch cultivations for production of recombinant proteins using the *AOX1 *expression system consist of three or four distinct phases [17-19]. The generation of biomass is achieved by growing the cells in two phases using glycerol as carbon substrate [17,19]. Initially, the cells are grown on glycerol in batch mode. For this phase, a maximum level of 40 g L^-1 ^glycerol is recommended due to potential inhibitory effects of elevated glycerol concentrations [19]. After consumption of glycerol, a glycerol-limited feeding phase follows until the desired level of biomass is reached. A glycerol-limited feeding phase has been also considered necessary for derepression of the *AOX1 *promoter, preparing cells for efficient production during the methanol fed-batch phase [17]. After reaching the desired biomass concentration, methanol-feeding is initiated to start recombinant protein production [17]. In Mut^s ^cultivations it is recommended to keep the methanol concentration below 3 g L^-1 ^[19,20] and, independent of the phenotype, it has been advised to keep the methanol concentration below 4 g L^-1 ^[18]. A variety of different methanol feeding strategies have been applied based on either methanol-limiting or non-limiting conditions preventing or even allowing the occurrence of oxygen limitation [21,22]. Some protocols even advocate two-phase induction strategies [23,24]. Sometimes mixed (methanol-glycerol) feeding protocols are also suggested for a more smooth methanol adaptation [17] or decaying temperature profiles during the induction phase to reduce proteolysis and cell death [24]. A comparison of the performance of these different fed-batch strategies is difficult, in particular, when different recombinant proteins are produced. In contrast to these multi-phase protocols we propose a simple two-phase strategy that only includes a glycerol batch phase, to generate sufficient biomass, directly followed by the induction phase with a constant methanol concentration for the remainder of the cultivation, to achieve high-level production of the recombinant protein.

Thus, we have combined the first two phases of growth on glycerol into a single phase by using higher glycerol concentration than those recommended previously. Moreover, we used a lower salt concentration as usually suggested [19] to decrease the osmolarity of the medium. When aiming at low osmolarity, the nitrogen supply can be critical in high-cell density *Pichia *cultivations. To keep the osmolarity low, a low initial nitrogen concentration was chosen and ammonium hydroxide was employed for pH control, and thus, the majority of nitrogen was not added at the start, but during the cultivation (Fig. 1C).

In addition, we could show that a glycerol fed-batch phase is not required for adaptation to methanol. At the end of the glycerol batch phase after switching to methanol, cell growth is resumed after a short period of adaptation (~3–4 h, Fig. 1). This adaptation period is not longer than observed with a preceding glycerol-limited feeding phase [25,26].

Prevention of high methanol concentrations in *Pichia *cultivations has been advocated by many authors as this might lead to the generation of toxic products such as formaldehyde and hydrogen peroxide which may compromise cell viability and productivity [20,27]. Other groups noticed growth-inhibitory effects only at methanol concentrations >2% and recommended up to 1% methanol to induce foreign protein production [27,28]. As a compromise between the positive effect of high methanol concentrations on induction strength and the negative effect on cell viability emerges a methanol concentration of 0.4–0.6% as is reported in our study and also by other groups [29].

*P. pastoris *is an obligate aerobe, and thus, oxygen availability is of critical importance. In the past, it has been strongly advised to prevent oxygen limiting conditions in high-cell density *Pichia *cultures, either by reducing the methanol supply [20] or enriching the inlet air with pure oxygen. More recently, however, it has been shown that reduced oxygen supply may be even advantageous for the production of recombinant proteins using the *P. pastoris AOX1 *expression system [23,25,26,30]. This is in agreement with our observation that there is no need to enrich the inlet air with pure oxygen or reduce the methanol supply to prevent oxygen limiting conditions during the induction phase to reach high-level production of HBsAg (Fig. 1D).

In conclusion, a simple two-phase high-cell density fed-batch procedure composed of a glycerol batch and a constant methanol fed-batch phase based on robust FID *on-line *analytics is proposed that generates ~7 times more HBsAg than previously reported using *Pichia *based expression systems.

## Methods

### Strain and preparation of precultures

The Mut^S ^strain of *P. pastoris *GS115 carrying 8-copies of the *HBsAg *gene under the control of the *AOX1 *promoter has been described before [31]. A 1-L preculture was prepared in two stages as follows. First, a 20-mL pre-preculture was prepared using 20 μL of the stock culture which was grown at 30°C in an orbital shaker (Multitron II, Infors AG, Germany) at 150 rpm for 48 h. This preculture was used to inoculate a 1-L preculture that was grown for 17 h, to serve as inoculum for the bioreactor culture. Cultures were grown in basal medium, in baffled flasks whose volumes were ~5× the culture volume to permit adequate aeration. The basal medium containing glycerol (20 g L^-1^), yeast nitrogen base (13.4 g L^-1^) and biotin (400 μg L^-1^) was prepared in MilliQ water.

### High-cell density fed-batch cultivation, culture conditions and control

Cultivation was carried out in a 15 L BIOSTAT-C (B. Braun Biotech International, Germany) bioreactor interfaced with UBICON for data acquisition and control. A 1-L inoculum, prepared as described above, was transferred to the bioreactor containing 9 L growth medium. The growth medium contained per liter: glycerol, 95.2 g; potassium *di*-hydrogen phosphate, 9.4 g; yeast trace metal (YTM) solution, 1.14 g; ammonium sulfate, 15.7 g; magnesium sulfate *hepta*-hydrate, 1.83 g; calcium chloride *di*-hydrate, 0.28 g; and biotin, 0.4 mg. The YTM solution contained: potassium iodide, 207.5 mg L^-1^; manganese sulfate, 760.6 mg L^-1^; *di*-sodium molybdate, 484 mg L^-1^; boric acid, 46.3 mg L^-1^; zinc sulfate *hepta*-hydrate, 5.032 g L^-1^; ferric chloride *hexa*-hydrate, 12.0 g L^-1^; and sulfuric acid, 9.2 g L^-1^. Antifoam (Ucolub N115) was added manually to control foaming in the bioreactor. Temperature was maintained at 30°C and pH at 5.6 with 12.5% (v/v) NH_4_OH. Aeration rate of 5 L min^-1 ^was constant throughout the process. The stirrer speed was controlled between 100 to 1370 rpm aiming at DO concentration of 20% air saturation. After consumption of glycerol, indicated by an increase of the DO concentration, production of recombinant HBsAg was initiated by the addition of a methanol solution [98.6% (w/w) methanol and 1.4 % (w/w) YTM] to a final methanol concentration of 6 g L^-1^. For the remainder of the cultivation, the methanol concentration was kept at 6 g L^-1 ^in the culture medium based on *on-line *measured methanol concentrations determined from the methanol vapor in the off-gas using a FID (Ratfish Instruments, Germany). Based on the gas liquid phase equilibrium methanol concentrations were determined in the off-gas using two point calibrations directly before and after induction. The concentrations of oxygen and carbon dioxide in the exhaust gas were determined by paramagnetic and infrared exhaust gas analysis systems, respectively (Maihak, Hamburg, Germany).

### Determination of cell concentration

The cell concentration was determined by measuring the optical density (OD) at 600 nm, of suitably diluted cultures, using a Novaspec II spectrophotometer (Pharmacia LKB). For CDM determination, 1 mL aliquots of the culture broth were centrifuged (13,000 rpm for 15 min at room temperature using an Eppendorf microcentrifuge, model 5415C) in pre-weighed tubes, re-suspended in 50 mmol L^-1 ^phosphate buffer, pH 7.2, re-centrifuged and the resultant pellets vacuum-dried at 80°C (Heraeus Instruments, Vacutherm) to constant mass. CDM was measured in triplicates and averaged. Cell viability in aliquots withdrawn at different time points during the cultivation was assessed by flow cytometry as described before [30].

### Determination of glycerol concentration

Glycerol concentrations in culture samples were determined using glycerol test kits (Roche, Basel, Switzerland). Briefly, 1-mL culture samples were centrifuged (13,000 rpm for 15 min at 4°C) and the glycerol concentration in the supernatant was analyzed in duplicate.

### Preparation of total cell extracts

Samples corresponding to 100 OD units (1 mL) were transferred and pelleted by centrifugation at 13,000 rpm for 15 min. The cell pellet was washed using 1 mL 10 mmol L^-1 ^phosphate buffer, pH 7.2 and mixed with 0.6 g glass beads of 0.45 mm diameter suspended in 500 μL of lysis buffer [10 mmol L^-1 ^phosphate buffer, pH 8, 5 mmol L^-1 ^EDTA, 500 mmol L^-1 ^NaCl, 8% (v/v) glycerol]. The cells were lysed by 20 cycles of 1-min vortexing at maximum speed followed by 4–5 min chilling on ice. After the final cycle of vortexing and chilling, the lysate was transferred into a fresh tube. The glass beads were rinsed twice with lysis buffer and pooled with the initial lysate to obtain a final volume of 1 mL.

### SDS-PAGE and Western blot analyses

A 50 μL aliquot of this lysate was mixed with an equal volume of a modified SDS sample buffer [5% (w/v) SDS, 50% (v/v) β-mercaptoethanol, 0.5 mol L^-1 ^DTT, 3% (v/v) glycerol], vortexed for 1 min and boiled for 15 min and then analyzed on a 15% SDS-polyacrylamide gel (10 μL loaded per lane). To visualize polypeptide profiles of the lysates before and after induction, the gels (run at 150 V for 80 min) were stained using Coomassie colloidal blue silver [32]. For Western blot analysis, the separated proteins were electro-transferred to PVDF membranes (pre-treated with methanol and washed with transfer buffer) at 100 V for 90 min. The PVDF membrane with the electro-transferred proteins was blocked for 1 h at room temperature, in phosphate buffered saline containing 1% (v/v) Tween 20, 5% (w/v) skim milk and 2% (w/v) polyvinyl pyrrolidone. HBsAg was detected in the blot using a commercially available rabbit polyclonal anti-HBsAg antiserum (Acris Antibodies GmbH, Germany) in conjunction with anti-mouse IgG-alkaline phosphatase conjugate and BCIP/NBT substrate (Calbiochem, La Jolla, CA).

### Determination of protein concentration

Protein content of *P. pastoris *total cell extracts was estimated by Pierce bicinchoninic acid (BCA) method [33].

### Determination of soluble and total HBsAg

VLPs in the clarified extracts (soluble HBsAg) were determined using the particle-specific quantitative Hepanostika micro ELISA system (Hepanostika Ultra Elisa, Biomerieux, France). Prior to the assay, the total cell extracts were processed as follows. Tween 20 was added to the extract to a final concentration of 1% (v/v) followed by vortexing at maximum speed for 4 hours at room temperature, to permit maximal solubilization of the rcombinant HBsAg. The samples were centrifuged at maximum speed at room temperature for 10 min. The resultant supernatant was then diluted 20,000-fold with 0.1% (w/v) bovine serum albumin in phosphate buffered saline. This material was used in the Hepanostika micro ELISA. In-house HBsAg ranging in concentrations from 1–100 ng mL^-1 ^was processed in parallel. The in-house HBsAg standard was pre-calibrated against NIBSC reference (second international standard: code – 00/588).

Total HBsAg was determined by reversed phase-high performance liquid chromatography (RP-HPLC) as reported earlier [34]. For this, a 100-μL aliquot of the *P. pastoris *cell lysate was suspended in an equal volume of solubilization reagent [8% (w/v) SDS, 50% (v/v) β-mercaptoethanol, 1 mol L^-1 ^DTT] boiled for 15 min and clarified by centrifugation and filtration. A 50 μL aliquot of this clarified sample was analyzed on a 3 μm SUPELCOSIL™ LC-DB-18 column (3.3 cm × 4.6 mm), maintained at 70°C in HPLC column oven (CH – 500, Eppendorf, Germany), at a flow rate of 1 mL min^-1 ^using buffer A [0.15% trifluroacetic acid (v/v) in MilliQ water] and buffer B (2-propanaol:acetonitrile in 80:20 ratio) which were used to generate a gradient in which the percentage of buffer B changed with time as follows: 0–4 min, 45%; 4–10 min, 45–95%; 10–13 min, 95%; 13–14 min, 95–45%; 14–18 min, 45%. The entire process was performed using a Shimadzu liquid chromatography system equipped with an auto-injector (SIL-10 AD *VP*), UV-VIS detector (SPD-10A), pumps A and B (LC-10 AT *VP*) and controller (SCL-10 A *VP*). All the samples were analyzed at 214 nm.

### Electron microscopic analysis of HBsAg VLPs

Purified HBsAg VLPs were diluted with PBS-buffer (7 mmol L^-1 ^K_2_HPO_4_; 14 mmol L^-1 ^NaH_2_PO_4_; 130 mmol L^-1 ^NaCl, pH 7.2) to a final protein concentration of 50 μg mL^-1^, adsorbed for 2 minutes to a glow-discharged C-Formvar foil and negatively stained with 2% (w/v) uranylacetate, pH 4.5. Electron microscopic examination was done as described before [35]. An energy-filtered transmission electron microscope Libra 120 (Zeiss, Oberkochen, Germany) was used and zero-loss images were taken with a 2048 × 2048 CCD camera (Tröndle, Moorenweis, Germany) using an energy-width of 15 eV and an objective aperture of 90 μm.

## Competing interests

The authors declare that they have no competing interests.

## Authors' contributions

CG and AA carried out the cultivations. CG and SKN performed the HBsAg determinations. HL carried out the electron microscopy studies. TG implemented the methanol controller. AR provided the initial idea to combine the glycerol batch and fed-batch phase. SS and NK were responsible for the design and creation of the 8-copy HBsAg clone and for the initial draft of the manuscript. UR directed the work and prepared the final manuscript. All authors approved and read the final manuscript.
